# Cecum microbiota in rats fed soy, milk, meat, fish, and egg proteins with prebiotic oligosaccharides

**DOI:** 10.3934/microbiol.2021001

**Published:** 2021-01-14

**Authors:** Souliphone Sivixay, Gaowa Bai, Takeshi Tsuruta, Naoki Nishino

**Affiliations:** 1 Department of Animal Science, Graduate School of Life and Environmental Science, Okayama University, Okayama, Japan; 2 Department of Health Science and Social Welfare, Takahashi, Japan

**Keywords:** diet, gut, microbiota, protein, prebiotics

## Abstract

Diet is considered the most influential factor in modulating the gut microbiota but how dietary protein sources differ in their modulatory effects is not well understood. In this study, soy, meat (mixture of beef and pork), and fish proteins (experiment 1) and soy, milk (casein), and egg proteins (experiment 2) were fed to rats with cellulose (CEL) and raffinose (RAF); the microbiota composition and short-chain fatty acid concentration in the cecum were determined. Egg protein feeding decreased the concentration of acetic acid and the richness and diversity of the cecum microbiota. RAF feeding increased the concentrations of acetic and propionic acids and decreased the richness and diversity of the cecum microbiota. When fed with CEL, the abundance of *Ruminococcaceae* and *Christensenellaceae*, *Akkermansiaceae* and *Tannerellaceae*, and *Erysipelotrichaceae* enhanced with soy protein, meat and fish proteins, and egg protein, respectively. The effects of dietary proteins diminished with RAF feeding and the abundance of *Bifidobacteriaceae*, *Erysipelotrichaceae*, and *Lachnospiraceae* increased and that of *Ruminococcaceae* and *Christensenellaceae* decreased regardless of the protein source. These results indicate that, although the effect of prebiotics is more robust and distinctive, dietary protein sources may influence the composition and metabolic activities of the gut microbiota. The stimulatory effects of soy, meat, and egg proteins on *Christensenellaceae*, *Akkermansiaceae*, and *Erysipelotrichaceae* deserve further examination to better elucidate the dietary manipulation of the gut microbiota.

## 1. Introduction

The research exploring the health-promoting and disease-preventive effects of the gut microbiota is gaining momentum. Evidence has shown that the colonization and diversity of the gut microbiota are influenced by several factors including age, genetics, medications (antibiotics), geographical location, and mode of delivery at birth [1]. Diet is considered the most influential factor in shaping and modulating the gut microbiota [2]. Aside from conventional dietary fibers and non-digestible and fermentable oligosaccharides, macro- and micro-nutrients that facilitate the growth of gut microbiota and mediate healthy metabolic homeostasis have gathered scientific attention [3]. Any entities to which the gut microbiota sensitively respond are now considered valuable tools to exploit and develop new strategies to promote systemic health.

Substrates for gut fermentation are fundamentally non-digestible dietary components and host secretions mainly comprising mucin. Thus, highly digestible food proteins have been considered to have little influence on the shaping of the gut microbiota. However, a small amount (~12 g/day) of non-digestible protein and peptides may reach the large intestine [4],[5], being a potential factor affecting bacterial metabolism. Similar to short-chain fatty acids (SCFAs) produced mainly from carbohydrate fermentation, amines, ammonia, hydrogen sulfide, and phenolic and indolic compounds may be released during protein fermentation [4]. The amount of protein entering the large intestine can further increase with consumption of high-protein diets, which are commonly used for body weight control.

Several reports have demonstrated the distinctive modulation of the gut microbiota by dietary proteins. Zhu et al. [6] compared the effects of milk, soy, beef, pork, chicken, and fish proteins on the gut microbiota and classified these sources into three groups, namely, non-meat (milk and soy), red-meat (beef and pork), and white-meat (chicken and fish) proteins. Bai et al. [7] revealed differences in the effects of milk and soy proteins on the gut microbiota and their modulatory effects on the functions of raffinose (RAF) and fructo-oligosaccharides. The low digestibility of plant protein has often been mentioned to account for the difference, if any, between plant and animal proteins [5], but the effect on shaping the gut microbiota was also shown to differ between milk, meat, and fish proteins [6],[8]. Although most animal proteins contain adequate proportions of essential amino acids, each protein source is composed of various protein fractions, such as myofibrillar, sarcoplasmic, and stromal fractions, with varying composition, structure, and function [9]. This could be the reason behind gut microbial diversity. Although there exist studies on animal proteins, only few have examined the effect of egg protein on the gut microbiota. Recently, several reports have revealed a distinctive difference between the effects of egg and other proteins [10],[11]. Nevertheless, how dietary protein sources modulate the effects of dietary fiber and prebiotic oligosaccharides is yet to be clarified.

In the present study, two experiments were conducted using rats to examine whether soy, meat, fish, milk, and egg proteins affect the gut microbiota and exert modulatory effects on the function of prebiotic RAF. Gut microbiota were assessed using a high-throughput 16S rRNA gene amplicon sequencing, which is an upgrade from denaturing gradient gel electrophoresis and group-specific qPCR employed in our previous studies [7],[12]. The objective of this study was to gain further insight into the dietary factors that affect the gut microbiota and explore improved dietary manipulation of healthy metabolic homeostasis through the gut microbiota.

## 2. Materials and methods

### 2.1. Animals and diets

Procedures and protocols for the animal experiments were approved by the Animal Care and Use Committee, in accordance with the guidelines of the Advanced Science Research Center, Okayama University. Two experiments were performed using female Wistar rats (4-weeks-old) purchased from the Charles River Laboratories Japan, Inc. and housed individually in stainless steel cages in a temperature-controlled room. The diet was formulated (per kg) with 200 g protein (soy, meat, or fish protein in experiment 1; soy, casein, or egg protein in experiment 2), 444.5 g corn starch, 150 g sucrose, 50 g lard, 50 g rapeseed oil, 40 g mineral mixture (AIN-93G-MX), 10 g vitamin mixture (AIN-93-VX), 3 g L-cystine, 2.5 g choline bitartrate, and 50 g non-digestible but fermentable carbohydrate (cellulose or raffinose). Soy protein isolate (Fuji Oil Co. Ltd., Osaka, Japan), casein (Oriental Yeast Co. Ltd., Tokyo, Japan), and egg albumin (Nacalai Tesque Inc., Kyoto, Japan) were commercially available and used for diet formulation without any further processing. Meat (mixture of minced beef and pork) and fish (Pacific ocean perch fillet) were procured from retailers. After freeze-drying, the oils were removed by diethyl ether extraction for 24 h. All rats were exposed to the same amount of feed at sufficient levels; the amount was determined as 2% more than the lowest consumption recorded on the previous day. Water was available ad libitum.

After 4 weeks of diet feeding, the rats were sacrificed by carbon dioxide gas inhalation followed by cervical dislocation. Cecum contents were collected to analyze short-chain fatty acid (SCFA) levels and to extract bacterial DNA, which was subsequently used for 16S rRNA gene amplicon sequencing.

### 2.2. Cecum SCFA analysis

For SCFA determination, cecum contents were homogenized with phosphate-buffered saline and deproteinized using 50 g/L metaphosphoric acid. The supernatant was used for the analysis of SCFA concentration using a gas liquid chromatograph (GC-14A; Shimadzu, Kyoto, Japan) fitted with a glass capillary column (15 m × 0.53 mm) coated with modified polyethylene glycol terephthalic acid (TC-FFAP; GL Sciences, Tokyo, Japan). The temperature of the column oven was programmed at 80°C for the first 2 min and was then increased to 200 °C at a rate of 10 °C/min.

### 2.3. Bacterial DNA extraction and 16S rRNA gene amplicon sequencing

In brief, 0.2 g of cecum samples was used for bacterial DNA extraction as per the procedure of repeated bead beating plus column method [13]. The extracted DNA was purified using the mini DNeasy stool kit (Qiagen, Germantown, MD, USA).

Bacterial DNA was amplified by two-step polymerase chain reaction (PCR) to generate amplicon libraries for next-generation sequencing [14]. The primers targeting the V4 region of 16S ribosomal RNA (rRNA) genes (forward: 5′-ACACTCTTTCCCTACACGACGCTCTTCCGATCTGTGCCAGCMGCCGCGGTAA-3′; reverse: 5′-GTGACTGGAGTTCAGACGTGTGCTCTTCCGATCTGGACTACHVGGGTWTCTAAT-3′) were used for the first round of PCR. The PCR products were purified by electrophoretic separation on a 2.0% agarose gel using a Fast Gene Gel/PCR Extraction Kit (Nippon Genetics Co., LTD., Tokyo, Japan). The second round of PCR was carried out using adapter-attached primers. The second-round PCR products were purified in the same way as in the first round.

**Table 1.** Weight, short-chain fatty acid concentrations, and diversity and relative abundance of the microbiota in the cecum of rats fed soy, meat, and fish proteins with cellulose (CEL) and raffinose (RAF).

Item	Soy		Meat		Fish		SE	Wilcoxon test	
	CEL	RAF	CEL	RAF	CEL	RAF		P	F
Cecum (g/100 g body weight)	1.68	3.05	1.34	2.55	1.51	2.43	0.18	NS	**^4^
Short-chain fatty acids (mmol/g)								
Acetic acid	42.7	46.6	35.6	56.9	25.9	51.0	7.02	NS	*^4^
Propionic acid	2.94	3.77	3.21	5.10	3.03	4.53	0.34	NS	**^4^
Butyric acid	2.94	3.37	2.55	3.30	1.77	2.35	0.65	NS	NS
Microbiota									
Population (log_10_ copies/g)	13.1	12.8	13.2	12.4	12.9	12.6	0.23	NS	*^5^
Alpha diversity									
Chao 1	181	88.1	152	111	172	89.2	9.68	NS	**^5^
Shannon	5.29	4.05	4.66	4.48	4.76	4.33	0.16	NS	**^5^
Relative abundance (%)									
Actinobacteria	0.10	9.57	0.04	3.31	0.69	3.77	1.19	NS	**^4^
*Bifidobacteriaceae*	0.04	8.38	0.00	3.27	0.04	3.19	1.26	NS	**^4^
*Coriobacteriaceae*	0.00	1.09	0.00	0.00	0.00	0.10	0.37	NS	NS
*Eggerthellaceae*	0.06	0.08	0.03	0.04	0.63	0.46	0.07	**^1^	NS
Bacteroidetes	8.36	14.4	8.56	21.3	12.6	18.1	3.10	NS	*^4^
*Bacteroidaceae*	4.18	8.81	1.17	8.28	1.14	7.55	1.50	NS	**^4^
*Tannerellaceae*	4.18	5.59	7.39	13.1	11.4	10.6	2.35	*^2^	NS
Firmicutes	84.2	74.0	70.7	67.7	66.1	73.7	3.43	*^3^	NS
*Lactobacillaceae*	7.12	19.9	6.65	7.69	3.92	17.1	3.26	NS	**^4^
*Christensenellaceae*	2.51	0.04	1.92	2.20	3.64	0.46	0.73	NS	**^5^
*Clostridiaceae* 1	0.00	0.00	0.05	0.25	0.03	0.00	0.11	NS	NS
*Lachnospiraceae*	11.9	22.1	17.7	21.9	14.8	23.0	3.15	NS	*^4^
*Peptococcaceae*	1.88	0.48	2.01	1.14	3.46	2.02	0.48	NS	*^5^
*Peptostreptococcaceae*	0.05	0.43	0.56	0.45	0.45	0.06	0.22	NS	NS
*Ruminococcaceae*	57.3	12.0	37.3	23.2	33.2	11.2	5.36	NS	**^5^
*Erysipelotrichaceae*	2.60	18.2	3.78	10.6	5.89	19.5	1.64	NS	**^4^
Proteobacteria	0.45	1.96	1.10	2.19	2.39	1.87	0.70	NS	NS
*Burkholderiaceae*	0.39	1.91	0.87	1.92	1.83	1.75	0.66	NS	NS
Tenericutes	2.57	0.00	0.74	0.08	0.44	0.00	0.29	NS	**^5^
*Anaeroplasmataceae*	0.00	0.00	0.52	0.08	0.07	0.00	0.17	NS	NS
Verrucomicrobia	4.31	0.05	18.8	5.35	17.8	2.58	2.52	*^2^	**^5^
*Akkermansiaceae*	4.31	0.05	18.8	5.35	17.8	2.58	2.52	*^2^	**^5^

Phyla and families with a relative abundance of > 1% in at least one sample are indicated. P, effect of protein; F, effect of fiber; NS, not significant; *, P < 0.05; **, P < 0.01.

^1^ Soy^b^ Meat^b^ Fish^a^; ^2^ Soy^b^ Meat^a^ Fish^a^; ^3^ Soy^a^ Meat^b^ Fish^ab^; ^4^ Cellulose < Raffinose; ^5^ Cellulose > Raffinose

The purified amplicons were pair-end sequenced (2 × 250 bp) on an Illumina MiSeq platform at FASMAC Co., Ltd. (Kanagawa, Japan). Raw sequence data were analyzed using the Quantitative Insights into Microbial Ecology (QIIME version 1.9.0). The 250-bp reads were truncated at any site receiving an average quality score under 20. The truncated reads that were shorter than 225 bp were discarded. In primer matching, sequences showing overlaps longer than 200 bp were assembled. The final reads obtained after pair-end joining were grouped into operational taxonomic units (OTUs) at a 97% similarity threshold. The sequence data were analyzed and categorized from the phylum to the family level using the default settings of the Ribosomal Database Project classifier.

### 2.4. Statistical analyses

Data related to cecum weight, cecum SCFA concentration, diversity index, and relative abundance of major bacterial families among the cecum microbiota (where the proportion of the family in at least one sample was > 1.0%) were analyzed by the non-parametric Wilcoxon test to examine the effects of dietary proteins and indigestible carbohydrates. The microbiota data were also subjected to principal coordinate analysis (PCoA) to define assignment and clustering that explained the variations in the microbiota. Discriminant vectors with a Pearson correlation > 0.7 were considered significant. The non-parametric test was performed using JMP software (version 11; SAS Institute, Tokyo, Japan), and PCoA was carried out using Primer version 7 with the Permanova+ add-on (Primer-E, Plymouth Marine Laboratory, Plymouth, UK).

## 3. Results

### 3.1. Experiment 1

The cecum weight (tissue and content) and acetic and propionic acid concentrations were higher and the total population, Chao 1 index, and Shannon index of the cecum microbiota were lower in the rats fed RAF than in those fed CEL (Table 1). None of the parameters were affected by dietary protein sources.

*Ruminococcaceae* (33.2–57.3%) was the most abundant family in the cecum microbiota of the rats fed CEL, regardless of the dietary protein source. The second and third most abundant families were *Lachnospiraceae* (11.9%) and *Lactobacillaceae* (7.12%) in the rats fed soy-CEL and *Akkermansiaceae* (17.8–18.8%) and *Lachnospiraceae* (14.8–17.7%) in the rats fed meat-CEL and fish-CEL. The modulatory effect of dietary proteins was observed on the abundance of several families among the cecum microbiota; the relative abundance of *Tannerellaceae* and *Akkermansiaceae* increased in the rats fed meat and fish proteins, while that of *Eggerthellaceae* increased in the rats fed fish protein. Remarkable changes were reported in the cecum microbiota of the rats subjected to RAF feeding; the abundance of *Bifidobacteriaceae*, *Bacteroidaceae*, *Lactobacillaceae*, *Lachnospiraceae*, and *Erysipelotrichaceae* increased and that of *Christensenellaceae*, *Peptococcaceae*, and *Akkermansiaceae* decreased in the rats fed RAF. The three most abundant families were *Lachnospiraceae* (22.1%), *Lactobacillaceae* (19.9%), and *Erysipelotrichaceae* (18.2%) in the rats fed soy-RAF, *Ruminococcaceae* (23.2%), *Lachnospiraceae* (21.9%), and *Tannerellaceae* (13.1%) in those fed meat-RAF, and *Lachnospiraceae* (23.0%), *Erysipelotrichaceae* (19.5%), and *Lactobacillaceae* (17.1%) in the rats fed fish-RAF.

**Figure 1.**
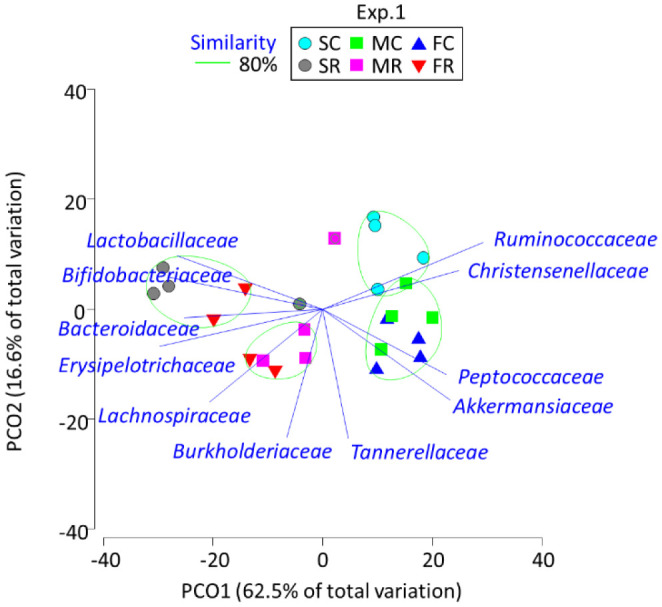
Principal coordinate analysis plot characterizing the cecum microbiota of the rats fed soy, meat, and fish proteins with cellulose and raffinose in experiment 1. SC, SF, MC, MF, FC, and FR indicate soy-cellulose, soy-raffinose, meat-cellulose, meat-raffinose, fish-cellulose, and fish-raffinose diet, respectively. Operational taxonomy units with Pearson's correlation > 0.7 are overlaid on the plot as vectors. The samples enclosed by the green lines belong to the same group (similarity level 80%).

The PCoA summed up the changes in the gut microbiota families in response to dietary protein sources and RAF feeding (Figure 1). Among CEL-treated rats, those fed soy protein formed a group separate from those fed meat and fish proteins. Rats fed soy-CEL were characterized by *Ruminococcaceae* and *Christensenellaceae*, while those fed meat-CEL and fish–CEL were characterized by *Peptococcaceae* and *Akkermansiaceae*. After RAF feeding, the separation between the rats fed soy and meat protein was retained, whereas that between the rats fed soy and fish became unclear. The rats fed soy-RAF were characterized by *Bifidobacteriaceae* and *Lactobacillaceae*, and those fed meat-RAF were associated with *Lachnospiraceae* and *Burkholderiaceae*.

### 3.2. Experiment 2

Consistent with the results of experiment 1, the cecum weight (tissue and content) and acetic and propionic acid concentrations were higher and the Chao 1 and Shannon indices of the cecum microbiota were lower in the rats fed RAF than in those fed CEL (Table 2). The cecum weight was higher and the cecum acetic acid concentration was lower in the rats fed egg protein.

*Ruminococcaceae* (34.8–42.8%) was the most abundant family in the cecum microbiota of the rats fed soy-CEL and casein-CEL, while *Erysipelotrichaceae* (48.7%) was the most enriched family in the rats fed egg-CEL. The second and third most abundant families were *Erysipelotrichaceae* (18.3–29.5%) and *Lactobacillaceae* (15.3–16.9%), respectively, in the rats fed soy-CEL and casein-CEL and *Ruminococcaceae* (19.5%) and *Lachnospiraceae* (11.5%), respectively, in those fed egg-CEL. Substantial changes were observed in the cecum microbiota after RAF feeding; the abundance of *Bifidobacteriaceae*, *Coriobacteriaceae*, *Lachnospiraceae*, and *Erysipelotrichaceae* increased and that of *Christensenellaceae*, *Clostridiaceae* 1, *Peptococcaceae*, *Peptostreptococcaceae*, and *Ruminococcaceae* decreased in the rats fed RAF. Many of the changes induced by RAF exposure in terms of cecum microbiota were the same between experiments 1 and 2; however, the increased abundance of *Bacteroidaceae* and *Lactobacillaceae* observed in experiment 1 was not reported in experiment 2, while the decreased abundance of *Clostridiaceae* 1 and *Peptostreptococcaceae* observed in experiment 2 was not reported in experiment 1. The three most abundant families were *Erysipelotrichaceae* (35.1%), *Lachnospiraceae* (27.6%), and *Lactobacillaceae* (12.4%) in the rats fed soy-RAF, *Erysipelotrichaceae* (41.1%), *Lachnospiraceae* (26.1%), and *Bifidobacteriaceae* (13.4%) in those fed casein-RAF, and *Erysipelotrichaceae* (53.5%), *Bifidobacteriaceae* (16.1%), and *Lachnospiraceae* (15.1%) in the rats fed egg-RAF.

**Table 2.** Weight, short-chain fatty acid concentrations, and diversity and relative abundance of the microbiota in the cecum of rats fed soy, casein, and egg proteins with cellulose (CEL) and raffinose (RAF).

Item	Soy		Casein		Egg		SE	Wilcoxon test	
	CEL	RAF	CEL	RAF	CEL	RAF		P	F
Cecum (g/100 g body weight)	2.48	4.78	2.54	4.08	3.93	4.80	0.29	*^1^	**^8^
Short chain fatty acids (mmol/g)								
Acetic acid	32.2	36.7	23.0	31.9	20.6	28.2	3.53	*^2^	*^8^
Propionic acid	5.23	6.10	3.83	7.65	5.72	8.28	0.69	NS	**^8^
Butyric acid	5.23	5.40	4.31	5.01	4.05	6.24	0.70	NS	NS
Microbiota									
Population (log_10_ copies/g)	12.5	13.8	13.2	13.2	12.6	13.6	0.22	NS	**^8^
Alpha diversity									
Chao 1	128	89.3	126	89.0	94.3	66.6	8.83	*^3^	**^9^
Shannon	4.35	3.79	3.94	3.53	3.26	3.05	0.13	**^4^	NS
Relative abundance (%)									
Actinobacteria	0.49	11.8	1.87	14.2	2.17	17.5	1.57	NS	**^8^
*Bifidobacteriaceae*	0.09	10.5	1.26	13.4	1.59	16.1	1.39	NS	**^8^
*Coriobacteriaceae*	0.00	0.95	0.00	0.00	0.00	1.07	0.27	*^1^	**^8^
*Eggerthellaceae*	0.36	0.34	0.60	0.77	0.57	0.32	0.14	*^5^	NS
Bacteroidetes	0.14	0.86	0.64	1.65	0.32	0.06	0.42	*^6^	NS
*Bacteroidaceae*	0.08	0.56	0.20	0.79	0.02	0.00	0.25	**^4^	NS
*Tannerellaceae*	0.06	0.30	0.44	0.85	0.30	0.06	0.20	*^6^	NS
Firmicutes	93.9	86.9	94.8	80.9	95.2	80.0	1.61	NS	**^9^
*Lactobacillaceae*	16.9	12.4	15.3	6.00	10.5	8.65	3.19	NS	NS
*Streptococcaceae*	5.10	3.59	0.44	1.24	0.15	0.79	0.51	**^2^	NS
*Christensenellaceae*	2.68	0.36	1.94	0.14	1.20	0.00	0.67	NS	**^9^
*Clostridiaceae* 1	0.06	0.00	0.53	0.00	0.93	0.21	0.23	NS	**^9^
*Lachnospiraceae*	5.25	27.6	9.18	26.1	11.5	15.1	2.42	NS	**^8^
*Peptococcaceae*	0.08	0.00	0.63	0.04	1.45	0.00	0.54	NS	**^9^
*Peptostreptococcaceae*	1.86	1.24	1.95	0.13	1.02	0.69	0.37	NS	**^9^
*Ruminococcaceae*	42.8	6.11	34.8	5.73	19.5	0.67	3.46	NS	**^9^
*Erysipelotrichaceae*	18.3	35.1	29.5	41.1	48.7	53.5	3.28	**^7^	NS
Proteobacteria	0.22	0.19	0.28	0.91	0.17	0.51	0.35	NS	NS
*Burkholderiaceae*	0.01	0.05	0.14	0.19	0.08	0.38	0.16	**^5^	NS
*Enterobacteriaceae*	0.16	0.11	0.11	0.68	0.05	0.13	0.28	NS	NS
Verrucomicrobia	4.63	0.26	2.06	2.38	2.17	1.94	1.35	NS	NS
*Akkermansiaceae*	4.63	0.26	2.06	2.38	2.17	1.94	1.35	NS	NS

Phyla and families with a relative abundance of > 1% in at least one sample are indicated.

P, effect of protein; F, effect of fiber; NS, not significant; *, P < 0.05; **, P < 0.01.

^1^ Soy^ab^ Casein^b^ Egg^a^; ^2^ Soy^a^ Casein^b^ Egg^b^; ^3^ Soy^ab^ Casein^a^ Egg^b^; ^4^ Soy^a^ Casein^a^ Egg^b^; ^5^ Soy^b^ Casein^a^ Egg^ab^; ^6^ Soy^b^ Casein^a^ Egg^b^; ^7^ Soy^b^ Casein^b^ Egg^a^; ^8^ Cellulose < Raffinose; ^9^ Cellulose > Raffinose

**Figure 2.**
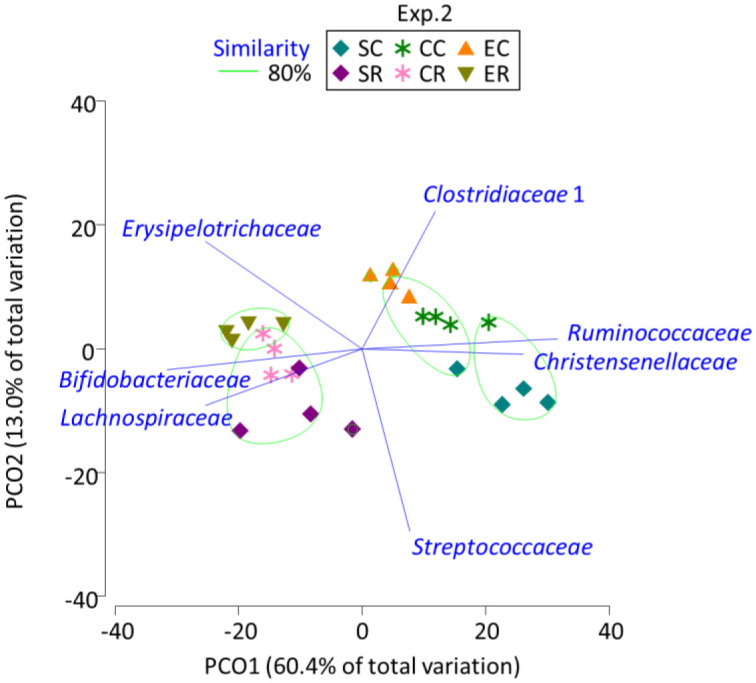
Principal coordinate analysis plot characterizing the cecum microbiota of the rats fed soy, casein, and egg proteins with cellulose and raffinose in experiment 2. SC, SF, CC, CF, EC, and ER indicate soy-cellulose, soy-raffinose, casein-cellulose, casein-raffinose, egg-cellulose, and egg-raffinose diet, respectively. Operational taxonomy units with Pearson's correlation > 0.7 are overlaid on the plot as vectors. The samples enclosed by the green lines belong to the same group (similarity level 80%).

The PCoA described separate groups for the rats fed egg protein and those fed casein and soy proteins, regardless of the fiber source (Figure 2). Following CEL administration, the rats fed soy protein appeared to be separated from those fed casein. In line with experiment 1 results, rats fed soy-CEL were characterized by *Ruminococcaceae* and *Christensenellaceae* and those fed soy-RAF and casein-RAF were related to *Bifidobacteriaceae* and *Lactobacillaceae*; the rats fed egg-RAF were characterized by *Erysipelotrichaceae*.

## 4. Discussion

The increase in the cecum weight of rats fed RAF may be attributed to the trophic effects of SCFAs. Although the concentration of butyric acid, which has a greater stimulatory effect on cell proliferation than acetic and propionic acids [15], did not increase, the increase of acetic and propionic acids after RAF feeding may have enlarged the cecum tissue weight in both experiments 1 and 2. The cecum weight also increased in rats fed egg protein, but this change was evident from the reduction in the acetic acid concentration in the cecum. Xia et al. [11] also found cecum enlargement in mice fed egg protein compared with those fed casein and soy protein. Although these authors ascribed cecum enlargement to the effect of resistant proteins, the data supporting this assumption were missing. Protein digestibility-corrected amino acid score and digestible indispensable amino acid score are the same for milk and egg proteins [9]; hence, an increase in cecum weight in egg protein-fed animals could not be accounted for by the digestibility of the protein. Regardless, Xia et al. [11] observed a reduction in α and β diversity of the cecum microbiota in response to egg protein feeding, which is consistent with our findings.

The increase in the cecum acetic acid concentration after RAF feeding can be ascribed to the increased abundance of *Bifidobacteriaceae*, an acetic acid producer, as well as the decrease in the Bacteroidetes/Firmicutes ratio, which was shown to be related to acetic acid concentration in the colon [18]. The increase in the cecum propionic acid concentration was also stably observed with RAF feeding; however, *Propionibacteriaceae*, a representative propionic acid producer, was quite low in relative abundance and hence not listed in the table. Although bacterial species could not be specified in this study, diverse bacteria could have been involved in the production of propionic acid through the succinate and acrylate pathways [16].

The finding that both Chao 1 and Shannon indices decreased after RAF feeding indicates that the diversity of the gut microbiota may be affected by prebiotic feeding. A loss in species diversity is generally related to disease symptoms, while increased richness in the gut microbiota is associated with positive health status [3]. However, certain supplements such as prebiotics could selectively enhance species with health-promoting potential, thereby lowering the richness and diversity of the gut microbiota. Although the reduction in Chao 1 and Shannon indices in rats fed egg protein as compared with those fed casein and soy protein in experiment 2 cannot be clearly explained, a similar decline in the richness and diversity owing to egg protein consumption was reported by Xia et al. [11].

The PCoA-based clustering and grouping may improve our understanding of the bacterial taxa involved in the diet-dependent structuring of the cecum microbiota; the growth of *Ruminococcaceae* and *Christensenellaceae* could be encouraged by soy protein feeding and that of *Akkermansiaceae* and *Tannerellaceae* by meat and fish proteins and *Erysipelotrichaceae* by egg protein. Prebiotic RAF feeding demonstrated a more robust influence on the cecum microbiota; the abundance of *Bifidobacteriaceae*, *Erysipelotrichaceae*, and *Lachnospiraceae* increased and that of *Ruminococcaceae* and *Christensenellaceae* decreased. An et al. [17] found that the abundance of *Ruminococcaceae* was higher after soy protein feeding than after casein and fish protein feeding. Zhu et al. [6] similarly found the stimulation of *Lachnospiraceae*, *Ruminococcaceae*, and *Lactobacillaceae* abundance following soy, meat, and fish protein feeding, respectively.

*Ruminococcaceae* and *Lachnospiraceae* are regarded as core commensal microbiota and, except that *Faecalibacterium prausnitzii*, a genus belonging to *Ruminococcaceae*, can exert anti-inflammatory functions [19], *Ruminococcaceae* and *Lachnospiraceae* are not referred to as the families associated with healthy metabolic homeostasis. The increased abundance of *Akkermansiaceae* may be considered a health-associated change after meat protein consumption because the level of *Akkermansia muciniphila* was shown to be positively correlated with the parameters involved in fatty acid oxidation and inversely associated with inflammatory markers and intestinal disorders, including inflammatory bowel disease [20],[21]. Furthermore, the abundance of *Christensenellaceae* negatively correlated with the pathological features of obesity, hypertriglyceridemia, and body mass index [22], and *Akkermansiaceae* and *Christensenellaceae* were both found to be the taxa of increasing abundance in the gut of centenarians [23],[24]. In contrast, the abundance of *Erysipelotrichaceae* was shown to be positively linked to lipidemic imbalance and hypercholesterolemia and reported to be increased in patients with colorectal cancer [25]. In the present study, RAF feeding stably stimulated the growth of *Erysipelotrichaceae* together with *Bifidobacteriaceae*; hence, although the increase in the abundance of *Erysipelotrichaceae* is regarded a biomarker of metabolic disorders, the reported increase in *Erysipelotrichaceae* after egg protein and prebiotic RAF feeding in this study can be considered a different outcome.

Although a number of protein effects on the gut microbiota and metabolism have been shown in this study, a short-term 4-week feeding with restricted data on the cecum contents could not indicate whether these changes may prevent or provoke chronic diseases and metabolic disorders. Considering the negative association of *Akkermansiaceae* and *Christensenellaceae* with intestinal disorders and lipidemic imbalance, long-term meat and fish feeding schedules with comprehensive analyses of histology, inflammation, and nutrient metabolism in the gut and liver are required. Moreover, the finding that egg protein feeding increased the cecum weight without an increase in the concentration of short-chain fatty acids may encourage the detailed examination of protein metabolites such as polyamines, hydrogen sulfide, and phenolic and indole compounds. Meanwhile, sex-based differences have been demonstrated with regard to the gut microbiota and metabolism [26]. Further studies are necessary to validate the several yet speculative interpretations presented in this study.

## 5. Conclusions

Soy, meat, fish, casein, and egg proteins differed in their modulatory effects in the shaping of the gut microbiota, although their effect on the gut microbiota was not more substantial than that of prebiotics. While the gut microbiota cannot be simply divided into taxa that can or cannot facilitate healthy metabolic homeostasis, the stimulatory effects of soy, meat, and egg proteins on levels of *Christensenellaceae*, *Akkermansiaceae*, and *Erysipelotrichaceae* deserve further investigation.
